# DNA watermarks: A proof of concept

**DOI:** 10.1186/1471-2199-9-40

**Published:** 2008-04-21

**Authors:** Dominik Heider, Angelika Barnekow

**Affiliations:** 1 Department of Experimental Tumorbiology, University of Muenster, Badestrasse 9, 48149 Muenster, Germany

## Abstract

**Background:**

DNA-based watermarks are helpful tools to identify the unauthorized use of genetically modified organisms (GMOs) protected by patents. *In silico *analyses showed that in coding regions synonymous codons can be used to insert encrypted information into the genome of living organisms by using the DNA-Crypt algorithm.

**Results:**

We integrated an authenticating watermark in the Vam7 sequence. For our investigations we used a mutant *Saccharomyces cerevisiae *strain, called CG783, which has an amber mutation within the Vam7 sequence. The CG783 cells are unable to sporulate and in addition display an abnormal vacuolar morphology. Transformation of CG783 with pRS314 Vam7 leads to a phenotype very similar to the wildtype yeast strain CG781. The integrated watermark did not influence the function of Vam7 and the resulting phenotype of the CG783 cells transformed with pRS314 Vam7-TB shows no significant differences compared to the CG783 cells transformed with pRS314 Vam7.

**Conclusion:**

From our experiments we conclude that the DNA watermarks produced by DNA-Crypt do not influence the translation from mRNA into protein. By analyzing the vacuolar morphology, growth rate and ability to sporulate we confirmed that the resulting Vam7 protein was functionally active.

## Background

Artificial DNA has been used for hiding messages or for data storage [1-5]. DNA-Crypt uses redundant ranges in the genetic code to introduce a watermark in a coding region. Amino acid codes are redundant so the watermark can be integrated by changing these DNA triplets. DNA-Crypt checks for synonymous codons in the genome and replaces the bases at the third position with a new base, which encodes parts of the watermark. The algorithm can be combined with other encryption algorithms like RSA, AES or Blowfish [6-9]. Mutations, which can occur will be corrected by DNA-Crypt itself using several mutation correction codes like the Hamming-code or the WDH-code [10]. An integrated fuzzy controller decides on a set of heuristics, whether to use a correction code or not for optimal performance. *In silico *studies using the Ypt7 gene of *Saccharomyces cerevisiae *showed that inserting these watermarks into a coding region did not affect the translation of proteins [11].

Searching for a homologous protein to mammalian Rab7 in *Saccharomyces cerevisiae*, Ypt7 was first discovered in 1992 by Wichmann *et al*. [12]. The Ypt7 gene encodes a 208 amino acid protein of 23.5 kDa [12]. It is involved in the homotypic vacuolar fusion and essential for the formation of the SNARE complexes at the vacuolar membrane [13,14]. In addition Ypt7 interacts with the HOPS-complex (homotypic fusion and protein sorting) and the Vam7 protein (Vam7p). A loss of Ypt7 leads to undocking of the HOPS-complex and Vam7p [15].

**Figure 1:**
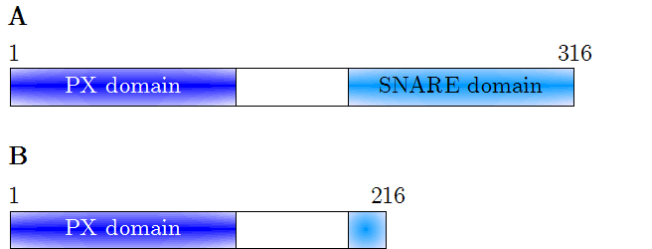
**Domain structure of the CG781 and CG783 Vam7 genes**. A: The Vam7 gene product of the parental CG781 strain. B: The gene product of the mutated CG783 strain. Because of the amber mutation at position 653 within the Vam7 sequence in CG783, 100 amino acids of the SNARE domain are missing [22].

The Vam7 gene was discovered in a screen for *Saccharomyces cerevisiae *mutants, which have defects in the vacuolar morphology [16]. The Vam7 gene encodes a 316 amino acid protein of 36.7 kDa. Strains lacking Vam7 or Ypt7 have various vesicular structures instead of distinct vacuoles [16]. Vam7p consists of two domains, the PX and the SNARE domain (Figure 1).

So far the PX domain has not been found in other SNARE proteins. It is thought to be necessary for the transport of Vam7p to the vacuolar membrane, whereas the SNARE domain is essential for the homotypic fusion [17,18].

The function of Vam7p in the sporulation processes of *Saccharomyces cerevisiae *has not been elucidated in detail yet, but it has been shown that ΔVam7 and ΔYpt7 strains are not able to produce spores [16,19]. In addition ΔVam7 strains exhibit a reduced proliferation rate in rich medium (YPD) [20].

**Figure 2:**
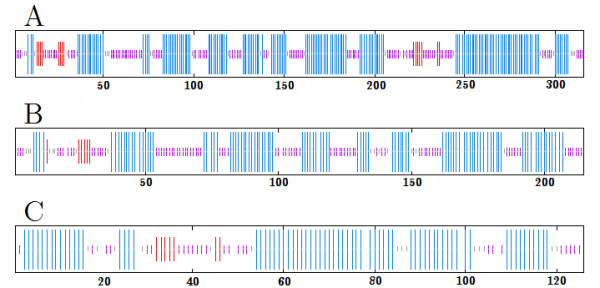
**Secondary structure consensus prediction using MLRC, DSC, PHD and PREDATOR [28-31]**. Alpha helices are blue, beta sheets are red and random coils are purple. A: Vam7p of CG781, B: inoperable Vam7 gene product of CG783, C: SNARE domain of wild type Vam7p.

For *in vivo *studies we used a *trp*^- ^mutant *Saccharomyces cerevisiae *strain (CG783) carrying a defective Vam7 gene (amber mutation at nucleotide 653 of 951 in the Vam7 gene) leading to incomplete vacuolar morphology (Figures 1, 2) [21]. In addition the CG783 strain is unable to sporulate [22].

As a control we used the parental CG781 strain, which carries an intact Vam7 gene (Table 1). Some times ago it was shown that transformation of CG783 with an intact Vam7 gene leads to normal vacuolar morphology and rate of sporulation (M. Kail unpublished data).

**Table 1:** Yeast strains

Strain	Genotype	Description
CG781	Ho ade1 trp1 ura1	parental strain of CG783 [22]
CG783	Ho trp1 ura1 spoT2-1	sporulation mutant [22]

## Results and Discussion

To investigate, whether the insertion of a watermark into the coding region of the Vam7 gene has an effect on the Vam7 protein, we produced a mutagenized Vam7 sequence, which we transferred into a yeast strain with an amber mutation within the Vam7 gene leading to an inoperable gene product.

**Figure 3:**
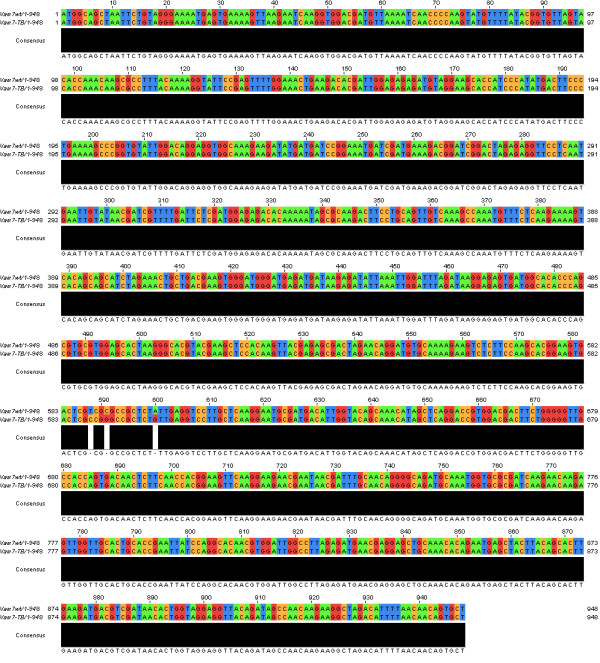
Pairwise sequence alignment of Vam7 and the watermarked Vam7-TB using ClustalW [23, 24].

The analysis of the watermarked DNA sequence with the DNA-Crypt fuzzy controller recommended not to use any correction code. The calculated mutation rate *φ *was 0.5833. A pairwise sequence alignment using ClustalW showed that the identity of Vam7 and the watermarked Vam7 sequence is 99.7% (Figure 3) [23,24]. The mutagenized base pairs are localized in the SNARE domain, which is thought to be essential for the homotypic fusion [17,18].

The integrated watermark did not influence the function of Vam7 and the resulting phenotype of the CG783 cells transformed with pRS314 Vam7-TB show no significant differences compared to the CG783 cells transformed with pRS314 Vam7.

**Figure 4:**
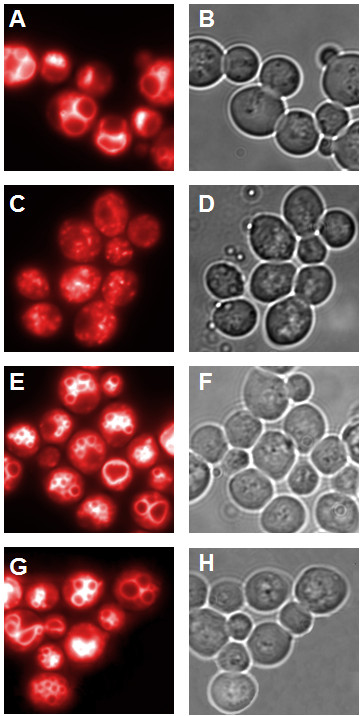
**Vacuolar morphology**. A,C,E,G: fluorescence microscopy; B,D,F,H: light microscopy; A,B: CG781 cells, C,D: CG783 cells, E,F:CG783 pRS314 Vam7 transformed cells, G,H: CG783 pRS314 Vam7-TB transformed cells (magnification 100×).

**Figure 5:**
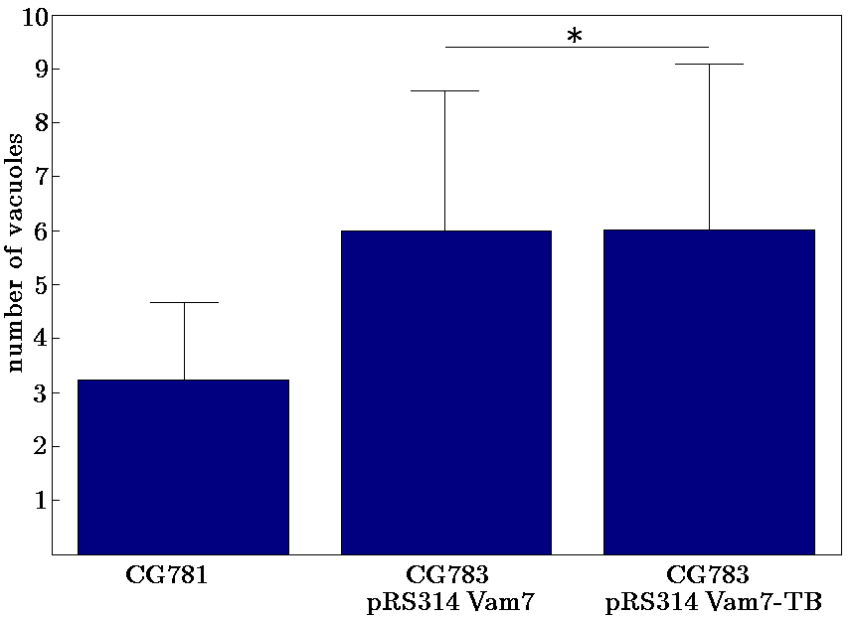
**Quantitative analysis of vacuoles per cell**. Three independent analyses were performed. All data show *p*-values < 0.01, except * *p *> 0.95.

The vacuolar morphology in both transformed strains was similar compared to the wild type strain (Figure 4). The number of vacuoles was slightly increased compared to the wild type strain (3.23 ± 1.44 vacuoles in CG781 cells, 6 ± 2.6 or 6.02 ± 3.07 in the pRS314 Vam7 or pRS314 Vam7-TB transformed strains respectively, but significantly different from CG783 cells, which contained various vesicular structures instead of a distinct vacuole (Figures 4, 5). As shown in Figure 5 CG783 cells transformed with pRS314 Vam7 and CG783 cells transformed with pRS314 Vam7-TB display no significant differences, which points to the fact that the insertion of the watermark has no influence to the resulting protein.

**Figure 6:**
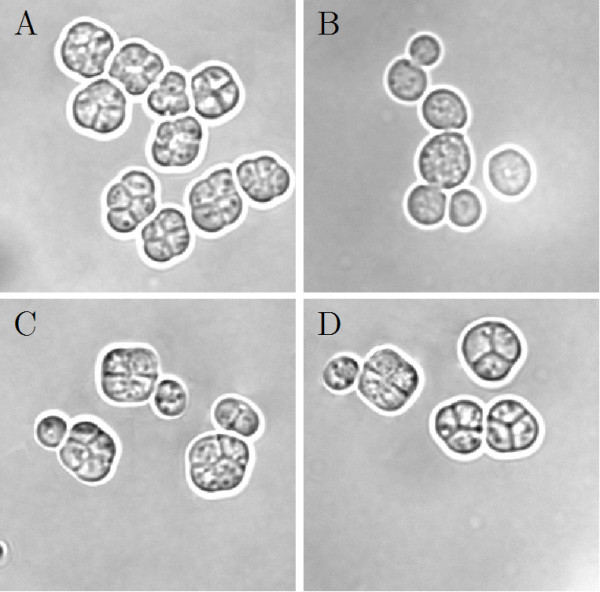
**Sporulation morphology**. A: Sporulation of the CG781 yeast strain; B: the sporulation deficient CG783 yeast strain; C: sporulation of the pRS314 Vam7 transformed CG783 yeast strain; D: sporulation of the pRS314 Vam7-TB transformed CG783 yeast strain (magnification 100×).

**Figure 7:**
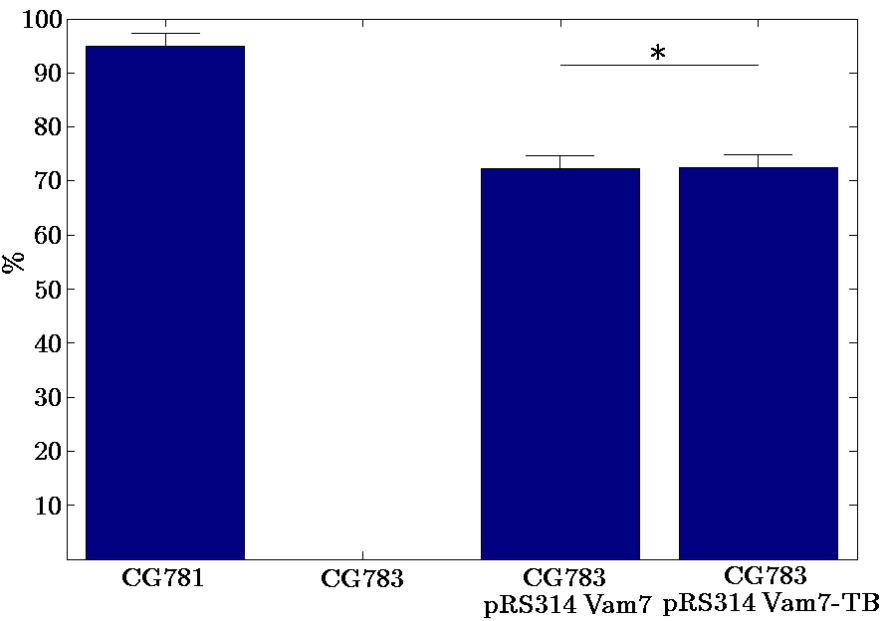
**Sporulation assay**. The percentage of sporulated yeast cells is shown. For experimental details see Methods. Five independent analyses were performed. All data show *p*-values < 0.01, except * *p *> 0.85.

In contrast to strain CG783, which is not able to sporulate most likely due to the lack of a functionally active Vam7 in CG783 cells, 72.4% ± 2.19 of the pRS314 Vam7 and 72.2% ± 2.59 of the pRS314 Vam7-TB transformed yeast cells formed spores. The spores of the transformed CG783 cells displayed the normal phenotype of CG781 spores (Figures 6, 7). Also in these experiments the insertion of the watermark did not result in an altered phenotype, comparing CG783 cells transformed with pRS314 Vam7 and CG783 cells transformed with pRS314 Vam7-TB.

**Figure 8:**
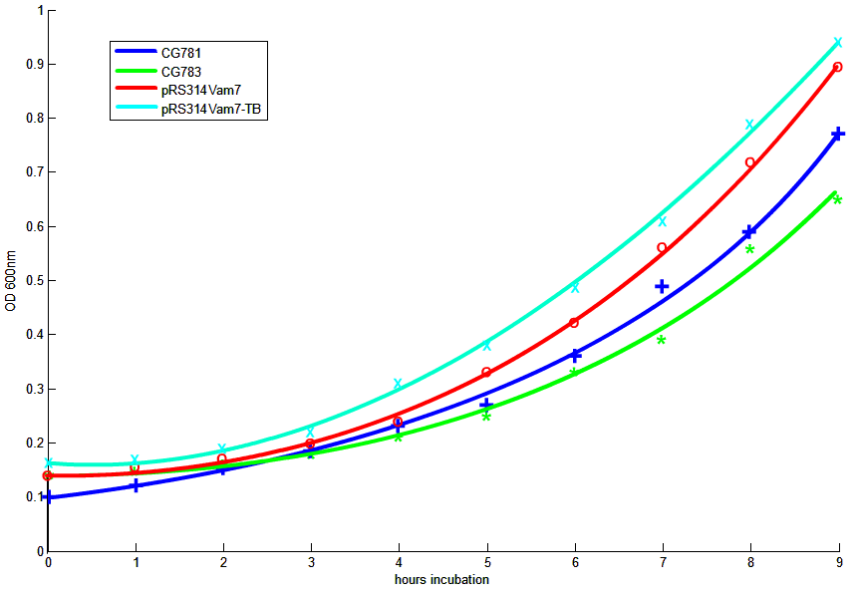
**Growth characteristics of strain CG781, CG783 and the pRS314 Vam7 and pRS314 Vam7-TB transformed CG783 strains**. Two independent experiments, where x is the corresponding mean with a standard deviation ≤ 0.03. All data show *p*-values < 0.05, except CG783 cells transformed with pRS314 Vam7 compared with CG783 cells transformed with pRS314 Vam7-TB (*p *> 0.65).

CG781 cells and the CG783 cells transformed with pRS314 Vam7 or pRS314 Vam7-TB, respectively, displayed a higher division rate compared to CG783 cells (Figure 8). The division rate (a) is calculated by

$$\frac{OD_{t9}}{OD_{t0}}=a^{10}$$

with measuring points *t*_*i*_, *i *= 0,.., 9.

In addition there are no significant differences comparing the division rates of the CG783 cells transformed with pRS314 Vam7 and the the CG783 cells transformed with pRS314 Vam7-TB (Figure 8).

Our aim was to prove that the insertion of the watermark 'TB' into the pRS314 DNA does not influence the expression of a functional Vam7 gene. By testing the morphology and several growth parameters we demonstrate that the cells containing pRS314 or pRS314 plus the watermark TB are indistinguishable from each other with respect to the parameters verified. The Vam7 negative and untransformed wild type cells were included in the experiments as controls.

Previously reported *in silico *studies using the Ypt7 genes of *Saccharomyces cerevisiae *demonstrated that using watermarks in coding regions did not influence the resulting protein [11]. In this paper we show for the first time that the *in silico *results could be confirmed *in vivo *by analyzing living yeast cells.

## Conclusion

To our knowledge the insertion of watermarks into eukaryotic cells has not been reported so far. Only storage of information in bacteria has been published [4,5]. As prokaryotic and eukaryotic cells show very different complexities e. g. on the levels of transcription, translation or compartmentalization it is very important to test and prove the application of DNA watermarks into coding regions of living eukaryotic cells. And this to our knowledge was successfully done for the first time in the experiments which we report on in this manuscript. Our DNA-Crypt algorithm clearly represents advances compared to the algorithms reported by Wong et al. or Arita and Ohashi. It permits the use of several mutation detection and correction codes, like the Hamming-code or the WDH-code and binary encryption algorithms like AES, Blowfish or RSA [6-10]. Further it provides the use of one-time pads [9]. Additionally the DNA-Crypt algorithm allows an increased amount of information per base and further has an integrated fuzzy controller that recommends whether to use a specific correction code or not [11].

Although, for reasons of economy, we inserted only 'TB' into the Vam7 gene more than two letters could be integrated without any effect to the resulting protein, because DNA-Crypt only produces silent mutations. To introduce longer watermark sequences more expensive synthesis of oligonucleotides and extended mutagenesis procedures would have to be performed. Nethertheless the resulting observations would be the same based on the fact that DNA-Crypt only produces silent mutations. Our experiments using the Vam7 gene are a proof of concept for introducing DNA watermarks into coding regions and can be generalized for other proteins.

The use of DNA watermarks in non coding or regulatory sequences will be subject to further examinations.

## Methods

### Watermark design

We wanted to encrypt the initials of our institute TB (Tumorbiology), translated the initials in a binary code and the binary sequence into a DNA sequence. Although we used a translation code, which slightly differs to the standard one used in DNA-Crypt, the binary encoding table is the same as used in Heider and Barnekow 2007 (Table 2) [11]. Moreover we scanned manually for the best location within the DNA sequence of Vam7 for integrating this watermark, which starts at position 588 within the wild type DNA sequence of Vam7 because of cost-benefit equation [11].

**Table 2:** Binary encryption table for the english alphabet

letter	binary	letter	Binary
A	00000_2_	N	01101_2_
B	00001_2_	O	01110_2_
C	00010_2_	P	01111_2_
D	00011_2_	Q	10000_2_
E	00100_2_	R	10001_2_
F	00101_2_	S	10010_2_
G	00110_2_	T	10011_2_
H	00111_2_	U	10100_2_
I	01000_2_	V	10101_2_
J	01001_2_	W	10110_2_
K	01010_2_	X	10111_2_
L	01011_2_	Y	11000_2_
M	01100_2_	Z	11001_2_

The inserted DNA watermark sequence is

*TB *→ 1001100001_2 _→ *CGCTG*

### DNA-Crypt fuzzy controller

We analyzed the watermark sequence with the DNA-Crypt fuzzy controller with standard settings. The life time was set at 1000 cycles [11,25]. The DNA-Crypt fuzzy controller recommends on a set of heuristics and three input dimensions, the individual mutation rate, the length of the watermark sequence and the life time of the watermark, which is represented in the number of generations the watermark is maintained, whether to use a mutation correction code or not [11].

#### Site-directed mutagenesis

To introduce the watermark into the DNA sequence we used a modified site-directed mutagenesis protocol with the pBluescript SKII plasmid (Stratagene, Amsterdam, The Netherlands) carrying the wild type Vam7 gene of *Saccharomyces cerevisiae*.

The modified site-directed mutagenesis was performed with 5, 20 and 50 ng of plasmid DNA using the following incubation mixture:

5 *μl *10× Pfx buffer (Invitrogen, Karlsruhe, Germany),

125 ng Vam7 – forward primer,

125 ng Vam7 – reverse primer,

0, 6 *μl *25 mM dNTPs,

1 *μl *50 mM *MgSO*_4_,

1 *μl *Platinum Pfx polymerase (Invitrogen, Karlsruhe, Germany),

ad. 50 *μl *A. bidest

The annealing temperature was 54°C for one minute and the elongation temperature 68°C for 6.5 minutes. We run 12 cycles in the PCR.

Vam7 – forward primer:

5'-CACGGAAGTGACTCGCCGGGCCGCTCTGTTGAGGTCCTTGCTC-3'

Vam7 – reverse primer:

5'-GAGCAAGGACCTCAACAGAGCGGCCCGGCGAGTCACTTCCGTG-3'

The mutagenesis was confirmed by sequencing with the M13 primer 5'-GTAAAACGACGGCCAGT-3'.

#### Subcloning

The mutagenized Vam7 insert in pBluescript SKII was subcloned into the pRS314 shuttle vector (Stillman D.J. 1993), which carries a tryptophane selection marker, using SacI/KpnI restriction enzymes (New England Biolabs, Frankfurt, Germany) and the T4 DNA-ligase (Fermentas GmbH, St. Leon-Rot, Germany).

#### Transformation of yeast

The yeast strain CG783 was transformed using the lithium acetate method and grown on SD -trp plates [26].

#### Fluorescence microscopy

The fluorescence stain FM4-64 was used to visualize the vacuolar membrane [27]. 5 ml of medium (SD or YPD) were inoculated with an overnight culture, to obtain an *OD*_600_0.2 – 0.3 and incubated for 2 to 3 hours at 30°C at 220 rpm on a shaker. 1 ml of the culture was centrifuged in a microfuge and suspended in 50 *μl *fresh medium containing 30 *μM *FM4-64. The cells were incubated for 15 minutes in a thermo mixer and then washed with 500 *μl *PBS. After centrifugation with 8000 × g for 3 minutes the pellet was suspended in 50 *μl *fresh medium. Now the cells were incubated at 30°C and 220 rpm for 1 to 4 hours. After centrifugation in a microfuge, the cells were suspended in 50 *μl *PBS and 1.5 to 3 *μl *of cells were used for fluorescence microscopy with 100× magnification (Leitz DIAPLAN and Photometrics Sensys).

#### Sporulation assay

5 ml of pre-sporulation medium (containing 2% potassium acetate, 1% yeast extract and 2% peptone (tryptic digested) were inoculated with 50 *μl *of an overnight culture. The cells were incubated at 30°C and 220 rpm overnight and then centrifuged at 3500 × g. After washing with 5 ml *H*_2_*O *the cells were suspended in 5 ml sporulation medium (containing 0.3% potassium acetate) and then transferred to an 100 ml Erlenmeyer flask containing 20 ml sporulation medium. After incubating for three to five days at 30°C and 220 rpm the spores were counted using a microscope with 100× magnification (Leitz DIAPLAN and Photometrics Sensys).

#### Growth characteristics

The growth characteristics of CG781, CG783 and the pRS314 Vam7 and pRS314 Vam7-TB transformed CG783 cells were analyzed by measuring optical densities at 600 nm every 60 minutes for 9 hours (Pharmacia LKB Novaspec II).

## Authors' contributions

DH, conception, structure predictions, sequence alignments, mutagenesis, transformation of yeast cells, microscopy, sporulation assay, growth characteristics, figure preparation, manuscript preparation AB, conception, design, manuscript preparation, coordination, research funds collection. The authors read and approved the final manuscript.
